# Highly efficient concentration of lenti- and retroviral vector preparations by membrane adsorbers and ultrafiltration

**DOI:** 10.1186/1472-6750-11-55

**Published:** 2011-05-20

**Authors:** Katrin Zimmermann, Oliver Scheibe, Andreas Kocourek, Jutta Muelich, Elke Jurkiewicz, Alexander Pfeifer

**Affiliations:** 1 Institute of Pharmacology and Toxicology, Biomedical Center, University of Bonn, Sigmund-Freud-Strasse 25, 53105 Bonn, Germany; 2 Sartorius Stedim Biotech GmbH, August-Spindler-Strasse 11, 37079 Goettingen, Germany; 3 PharmaCenter Bonn, University of Bonn, Bonn, Germany

## Abstract

**Background:**

Lentiviral vectors (LVs) can efficiently transduce a broad spectrum of cells and tissues, including dividing and non-dividing cells. So far the most widely used method for concentration of lentiviral particles is ultracentrifugation (UC).

An important feature of vectors derived from lentiviruses and prototypic gamma-retroviruses is that the host range can be altered by pseudotypisation. The most commonly used envelope protein for pseudotyping is the glycoprotein of the Vesicular Stomatitis Virus (VSV.G), which is also essential for successful concentration using UC.

**Results:**

Here, we describe a purification method that is based on membrane adsorbers (MAs). Viral particles are efficiently retained by the anionic exchange MAs and can be eluted with a high-salt buffer. Buffer exchange and concentration is then performed by utilizing ultrafiltration (UF) units of distinct molecular weight cut off (MWCO). With this combined approach similar biological titers as UC can be achieved (2 to 5 × 10^9 ^infectious particles (IP)/ml). Lentiviral particles from small starting volumes (e.g. 40 ml) as well as large volumes (up to 1,000 ml) cell culture supernatant (SN) can be purified. Apart from LVs, vectors derived from oncoretroviruses can be efficiently concentrated as well. Importantly, the use of the system is not confined to VSV.G pseudotyped lenti- and retroviral particles and other pseudotypes can also be purified.

**Conclusions:**

Taken together the method presented here offers an efficient alternative for the concentration of lenti- as well as retroviral vectors with different pseudotypes that needs no expensive equipment, is easy to handle and can be used to purify large quantities of viral vectors within a short time.

## Background

Lentiviral vectors (LVs) are versatile tools for molecular medicine and gene therapy [1]. They are able to integrate their viral genome into both dividing and non-dividing cells [2,3]. Most LVs presently used are based on the human immunodeficiency virus type 1 (HIV-1), which is the most extensively studied lentivirus [1]. In addition, lentivectors have been derived from a variety of other lentiviruses (e.g. simian, equine and feline lentiviruses) [4].

Lenti- as well as spuma- and oncoretroviruses belong to the large family of *Retroviridae *[5]. Retroviruses are - besides adenoviruses - the most widely used vectors in gene therapy clinical trials (http://www.wiley.co.uk/genmed/clinical). They were also the first vectors that have been applied in gene therapy. Most oncoretroviral vectors are based on the Moloney murine leukemia virus (MoMLV) [6]. The wild type MoMLV and retroviral vectors carrying the original MoMLV envelope proteins transduce only cells that express the ecotropic receptor, i. e. rodent cells [7,8]. However, oncoretroviral vectors are sensitive to environmental conditions and lose their infectivity relatively quickly [9,10] resulting in a loss of infectious particles (IP) during purification [11]. Lenti- as well as retroviral vectors can also be pseudotyped with different envelope proteins from other viruses resulting in different characteristics of the vectors, either regarding their surface charge, physical stability and/or biological activity [11]. Pseudotyping of LVs or oncoretroviral vectors with VSV.G broadens the host range by enabling them to enter the cells via receptor mediated endocytosis [12]. Furthermore, VSV.G-pseudotyped viral vectors were shown to be more stable and can be concentrated by ultracentrifugation (UC) without a significant loss in titer [13,14].

The most commonly used retro- as well as lentiviral vector systems are based on a split genome approach that provides the viral genes necessary *in trans *for production of viral particles in the helper/packaging cells [15,16]. The viral particles are released into the cell culture supernatant (SN) and can be easily harvested without need to disrupt the cells. Presently, the most common method to concentrate these viral particles from cell culture SN is UC [11,17]. In addition, different methods have been studied to purify lenti- and retroviral vectors including chromatography, ultrafiltration (UF) and coprecipitation with salts and/or polymers [11,18,19].

Here, we analyzed a purification system (LentiSELECT, Sartorius Stedim Biotech, Goettingen, Germany) that combines membrane adsorption and UF for the concentration of VSV.G pseudotyped lenti- as well as retroviral particles. The method used is based on binding of the viral particles to an anionic exchange membrane adsorber (MA) followed by UF of the eluted purified particles. Different volumes of cell culture SN, up to 1,000 ml, can be purified and the resulting recovery rates are reproducible and comparable to those achieved by UC. Furthermore, the method was also successfully applied for the purification of lentiviral particles without envelope proteins and retroviral particles with an ecotropic envelope, demonstrating the broad range of this application.

## Results

### Purification of lentiviral particles by using membrane adsorbers

**Figure 1:**
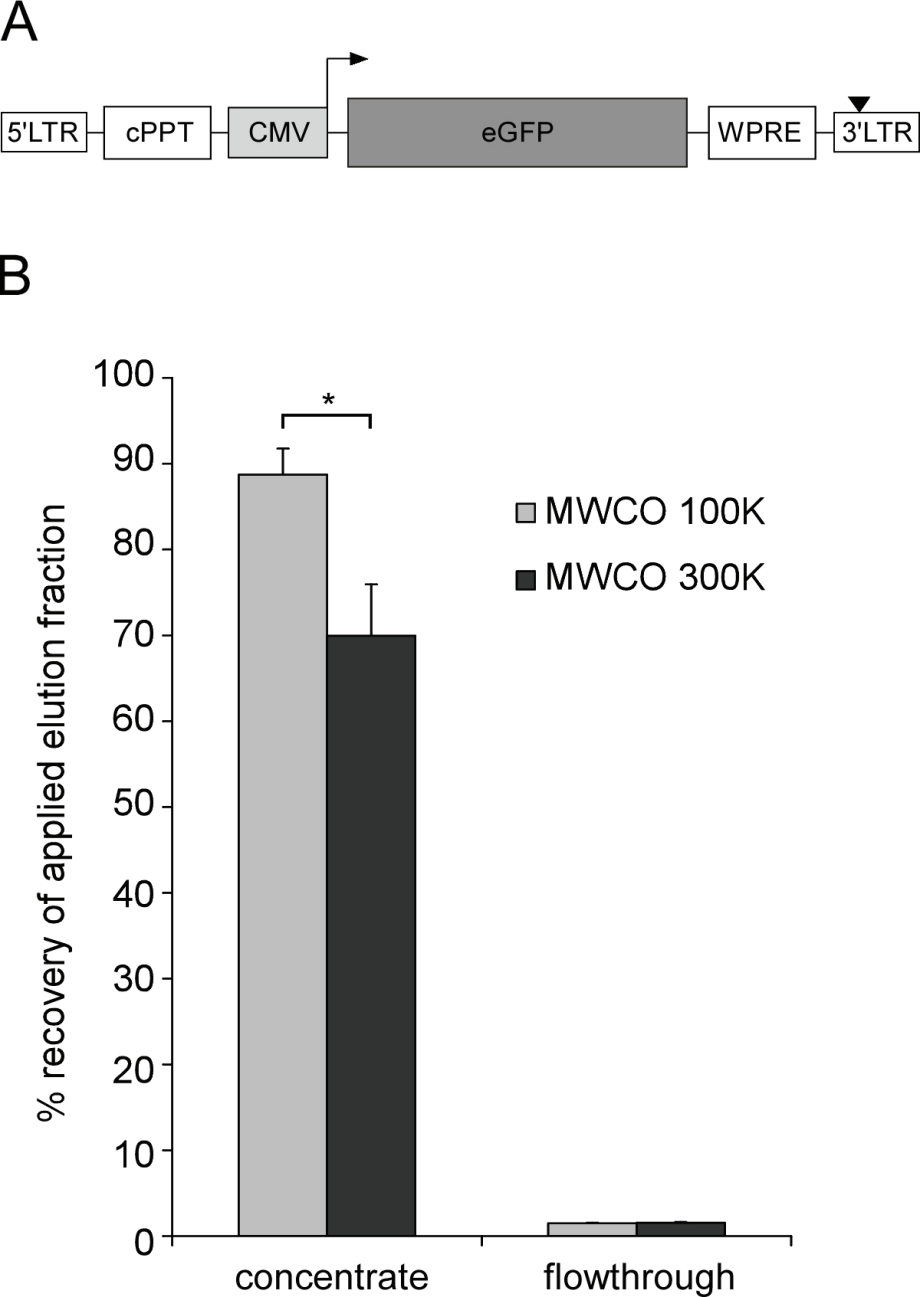
**Lentiviral vector construct and use of UF units with different MWCOs for concentration of LVs**. **(A) **Schematic representation of lentiviral vector used. eGFP, enhanced green fluorescent protein; CMV, promoter from cytomegalovirus; WPRE, posttranscriptional regulatory element of woodchuck hepatitis virus; cPPT, central polypurine tract; LTR, long terminal repeat; black triangle, mutation in 3' LTR, leading to self inactivation (SIN vector). **(B) **Comparison of Vivaspin20 UF units with different MWCOs: 1,000 ml of cell culture SN were purified using the large MAs. The elution fraction was desalted with Vivaspin20 UF units with MWCO of 100,000 (100 K) or 300,000 (300 K). The biological titer was determined by FACS-analysis and the recovery rate (%) was calculated. n = 4, mean + SEM. * 0.05 ≥ p > 0.01.

For purification of LVs, we initially focused on VSV.G pseudotyped lentiviral particles, because they are the most widely applied type of lentivectors. We used HIV-derived LVs carrying a CMV promoter-driven eGFP expression cassette [20] (Figure 1A). Viral particles were generated by transient transfection of HEK 293T cells. The cell culture SN was harvested and applied to anionic exchange MAs (LentiSELECT, Sartorius Stedim Biotech, Goettingen, Germany) to bind and enrich for virus particles. Samples of each purification step were then analyzed to quantify the amount of IPs by transduction of HEK 293T cells and FACS analysis.

After loading of the lentiviral particles to the MA and washing, retained viral particles were eluted with high salt elution buffer (1 M sodium chloride). Given the potential detrimental effect of high salt on the functionality of the viral particles, we investigated the impact of the hypertonic environment on viral infectivity. Incubation of the eluted LVs in the high salt buffer for one hour on ice resulted in a significant reduction of infectivity by 16.0 ± 6.4%. Within 2 hours 22.1 ± 6.4% of IPs were lost (Additional file 1, Figure S1). To prevent loss of infectivity a buffer exchange step was included to recover the viral particles in a buffer with physiological salt concentration (e.g. Hank's buffered salt solution (HBSS)). Buffer exchange as well as further concentration of the viral particles was achieved by using UF columns. The membranes of these columns have pores of distinct size that can retain viral particles, whereas smaller molecules and particle fragments can pass through the pores. Vivaspin20 UF units with 100 K or 300 K MWCOs were compared that can accommodate up to 20 ml of MA eluate (Figure 1B). In both cases, only 1.5 ± 0.1% of the viral particles were detected in the flowthrough. Although UF units with a MWCO of 300 K require less centrifugation time than the 100 K units due to the larger pore size (data not shown), the 100 K unit showed significant higher recovery of the viral particles (recovery rate of 88.7 ± 3.1%). In summary, the Vivaspin20 UF unit with a MWCO of 100 K is suitable for rebuffering, purifying as well as concentrating of the lentiviral particles after elution from the MA.

### Purification of lentiviral particles from small starting volumes

**Figure 2:**
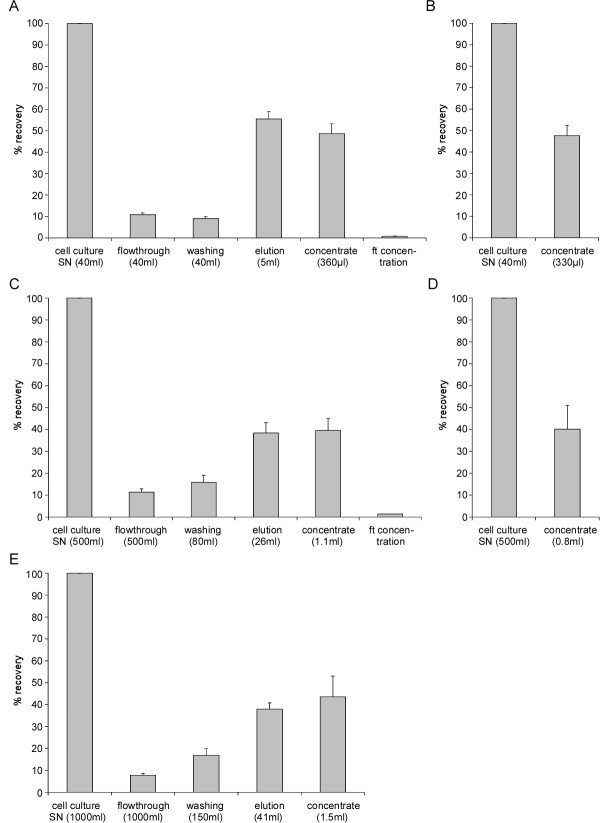
**Purification of LVs from different starting volumes**. LVs were purified from 40 ml cell culture SN by using the small MA/UF setup **(A) **or UC **(B)**; from 500 ml of starting volume by application of the large MA/UF combination **(C) **or UC **(D) **and from 1,000 ml of cell culture SN and use of two serially connected large MAs and UF **(E)**. IP/ml were determined via FACS-analysis and the recovery rate (%) was calculated. *n = 7 (A), n = 3 (B); n = 6 (C), n = 3 (D), n = 3 (E). mean + SEM.*

Importantly, the use of anionic exchange MAs should allow for the purification of lentiviral particles from small as well as large starting volumes. First, we tested a small volume system (LentiSELECT40), which can be used for up to 40 ml starting solution. The viruses are eluted by applying 4 ml elution buffer. Starting with 1.2 × 10^9 ^± 1.8 × 10^8 ^IPs in the cell culture SN 55.5 ± 3.4% of the viral particles applied were recovered from the MA (Table 1 and Figure 2A). Approximately 11 and 9% of virus was lost in the flowthrough and the wash fraction, respectively (Figure 2A). Subsequently, the eluate was loaded on the UF unit and the final volume of the retentate was adjusted to ~360 μl (Table 1). Overall, the final recovery rate of the small volume MA/UF combination was 48.7 ± 4.6% with 5.8 × 10^8 ^IPs in the concentrate (Table 1 and Figure 2A). Hence, a 4.5 fold concentration was achieved with the MA and a further 13 fold concentration was obtained by UF (Table 1). Taken together, the combination of anion exchange resin and UF unit resulted in a 59 fold enrichment of lentiviral particles from cell culture SN to concentrate. High enrichment was also seen in Westernblot analysis by using antibody against the lentiviral capsid protein p24 (Additional file 2, Figure S2).

**Table 1:** Comparison of different methods for purification of LVs.

	SN			elution			concentrate		
	
purification method	volume [ml]	IP/ml (± SEM)	IP (± SEM)	volume [ml]	IP/ml (± SEM)	IP (± SEM)	volume [ml]	IP/ml (± SEM)	IP (± SEM)
MA/UF *(n = 7)*	40	3.0 × 10^7^ (± 4.7 × 10^6^)	1.2 × 10^9^ (± 1.8 × 10^8^)	4.7 (± 0.2)	1.3 × 10^8^ (± 2.2 × 10^7^)	6.2 × 10^8^ (± 9.5 × 10^7^)	355 (± 32)	1.8 × 10^9^ (± 4.0 × 10^8^)	5.8 × 10^8^ (± 1.2 × 10^8^)

UC *(n = 3)*	40	1.2 × 10^7^ (± 1.3 × 10^6^)	4.7 × 10^8^ (4.8 ± × 10^7^)		-		327 (± 18)	6.8 × 10^8^ (± 8.9 × 10^7^)	2.1 × 10^8^ (± 2.1 × 10^7^)

MA/UF *(n = 6)*	500	1.4 × 10^7^ (± 8.6 × 10^5^)	7.1 × 10^9^ (± 5.2 × 10^8^)	25.8 (± 0.2)	1.5 × 10^8^ (± 3.5 × 10^7^)	2.7 × 10^9^ (± 3.5 × 10^8^)	1064 (± 118)	2.7 × 10^9^ (± 3.7 × 10^8^)	2.8 × 10^9^ (± 5.0 × 10^8^)

UC *(n = 3)*	500	2.5 × 10^7^ (± 6.6 × 10^6^)	1.3 × 10^10^ (± 3.3 × 10^9^)		-		776 (± 152)	6.1 × 10^9^ (± 1.1 × 10^9^)	4.6 × 10^9^ (± 1.1 × 10^9^)

MA/UF *(n = 3)*	1000	1.8 × 10^7^ (± 4.9 × 10^6^)	1.9 × 10^10^ (± 5.2 × 10^9^)	40.6 (± 0.7)	1.7 × 10^8^ (± 4.8 × 10^7^)	7.0 × 10^9^ (± 1.9 × 10^9^)	1510 (± 220)	5.4 × 10^9^ (± 1.8 × 10^9^)	8.6 × 10^9^ (± 3.6 × 10^9^)

To compare the method presented here with a standard procedure, we used a well established UC protocol [21] consisting of an initial centrifugation at 61,700 g for 2 h, followed by loading the resuspended viruses on a sucrose cushion. After a second spin at 53,500 g for 2 h, the viral precipitate was resuspended in ~330 μl HBSS. Starting initially with 4.7 × 10^8 ^IPs in the cell culture SN 47.7 ± 4.8% of the IPs were recovered by UC (Table 1 and Figure 2B), which is not significantly different from the recovery rate obtained by use of the MA/UF combination (*p = 0.445*).

### Purification of lentiviral particles from large starting volumes

Next, we tested whether larger volumes of cell culture SNs can be processed with MAs. For this purpose, we used a larger MA (LentiSELECT500) that can accommodate up to 500 ml starting volume. Using this MA/UF setup 39.6 ± 5.5% (2.8 × 10^9 ^± 5.0 × 10^8 ^IPs in approximately 1.1 ml) of the LVs were finally recovered from the cell culture SN (Table 1 and Figure 2C). This corresponds to a 193 fold enrichment of viral particles in the concentrate compared to the starting solution. Considering the differences in the reduction in volume from SN to concentrate for the small and the large MA, the overall enrichments are not significant different.

Using UC to purify viral particles from the same starting volume of 500 ml of SN, a recovery rate of 40.1 ± 10.9% was achieved (Table 1 and Figure 2D). Again, no significant difference between the recovery rates obtained by the use of MA/UF setup and UC was observed (*p = 0.480*).

It should be noted that for such large volumes UC is a time-consuming procedure, because multiple centrifugation runs are necessary due to the limited capacity of the rotors. For 500 ml cell culture SN the standard UC protocol requires overall 4 runs each lasting 2 hours (3 times at 61,700 g with a loading volume of 180 ml per run and a final concentrating run at 53,500 g). In contrast, loading, washing and elution of the MAs and subsequent UF requires only around 3 h.

To test whether two large MA units can be serially connected for further upscaling, we directly coupled two units. To evaluate the efficiency of the serial MA set-up, 1,500 ml of cell culture SN were prepared and 1,000 ml were loaded on two serially connected MAs and 500 ml were loaded on a single MA. The flowthrough was collected stepwise in 50 ml fractions and the biological titer (in IP/ml) was determined, respectively (Additional file 3, Figure S3). The two set-ups showed increasing concentrations of viral vectors in consecutive flowthrough fractions. For the single MA around 10% of the initial viral load was in the flowthrough after adding approximately 420 ml of cell culture SN onto the MA (Additional file 3, Figure S3A). Similarly, for the two coupled MAs 10% of the loaded viral particles were found in the flowthrough after application of 850 ml of starting volume (Additional file 3, Figure S3B). Thus, the maximal loading volume is ~500 ml and ~1,000 ml for one unit and for two coupled units, respectively. In summary, a serial connection of two large MAs can be used to increase the volume of loading material to up to 1,000 ml of cell culture SN. Purification of 1,000 ml of cell culture SN with a two-MA-setup and UF (LentiSELECT1000) resulted in a recovery rate of 43.6 ± 9.4%, displaying an enrichment of almost 300 fold from starting volume to concentrate (Table 1 and Figure 2E). This is comparable to the enrichments achieved by the purification of smaller SN volumes (40 ml or 500 ml) with MA/UF considering the reduction in volume from SN to concentrate.

### Analysis of the purified lentiviral vectors

**Figure 3:**
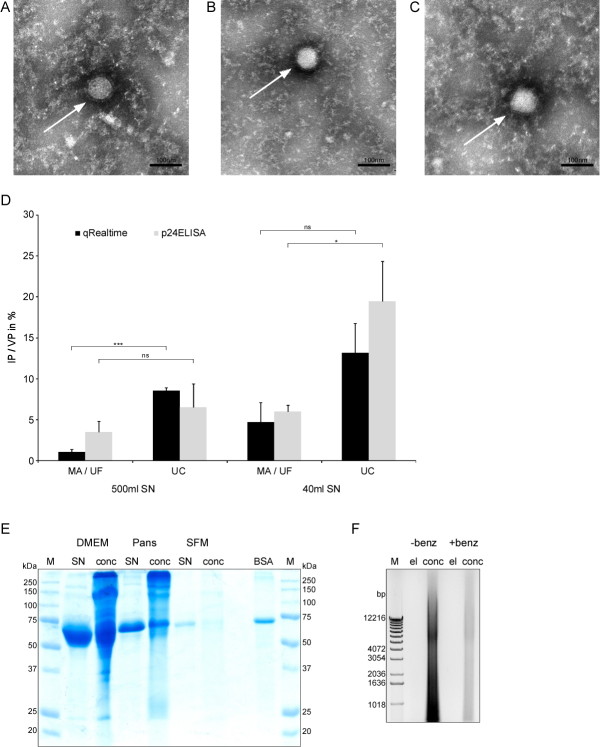
**Analysis of the purified LVs**. Cell culture SN was purified by UC or MA/UF. The concentrates were analyzed by electron microscopy: 40 ml SN, MA/UF **(A)**; 500 ml SN, MA/UF **(B)**; 500 ml SN, UC **(C)**. **(D) **Analysis of biological vs. physical titer (IP/VP) of different LV preparations: The biological titer was determined via FACS-analysis of transduced cells and the physical titer by use of a HIV-1 p24 Antigen ELISA (p24 ELISA) or QRTPCR (Realtime). Values were determined in triplet and/or use of different dilutions. Concentrates of the following purifications were analyzed: 500 ml SN, MA/UF (*n = 7*); 500 ml SN, UC (*n = 3*); 40 ml SN, MA/UF (*n = 4*); 40 ml SN, UC (*n = 7*). *mean+SEM*. * 0.05 ≥ p > 0.01; *** 0.001 ≥ p; ns - not significant. **(E), (F) **Analysis of protein and DNA content of LV preparations. After transfection of producer cells the medium was changed to 40 ml DMEM, supplemented with FCS (DMEM) or to serumfree medium Panserin 608 (Pans) or HyClone SFM4Megavir (SFM). Purification of LVs was performed by MA/UF. 10 μl of SN or concentrate (conc) as well as 5 μg pure BSA was loaded on the SDS-PAgel (coomassie blue stain). M, protein marker **(E)**. 500 ml of the same cell culture SN was each incubated at 37°C for 30 min either without addition of benzonase (-benz) or after adding 12.5 u/ml benzonase (+benz). The SN was purified using MA/UF, respectively. 5 μl of the elution fraction (el) or the concentrate (conc) were used for agarose gel electrophoresis. M, DNA marker **(F)**.

In order to analyze viral particle morphology and integrity, we applied electron microscopy. The size and appearance of particles purified by MA/UF were not different from those particles obtained by UC (Figure 3A-C).

A further parameter to compare different purification methods is the ratio of biological versus physical titer. This ratio indicates the percentage of viral particles that are actually infectious as compared to the overall viral particles (physical titer). We determined the biological titer by FACS analysis of transduced cells. The physical titer (viral particles/ml, VP/ml) was quantified by two methods that measure different constituents of the particles, i.e. the lentiviral capsid protein p24 or the viral genome as measured with quantitative Realtime PCR (QRTPCR). We analyzed the ratio of the biological and the physical titer of LV preparations derived from purifications using either MA/UF or UC with different starting volumes (Figure 3D). Overall the IP/VP ratios were higher for the low volume preparations independently of the purification method used and ca. 5 - 20% of the particles purified from low volumes were biologically active, whereas only 1 - 9% purified from larger volumes were infectious. These ratios are in the same range or even higher than those found in the literature where ratios between 0.1 to 1% were observed [22,23].

Comparison of the two purification methods revealed also considerable variations: The IP/VP ratios for UC are in the range of 7 - 20% and 9 - 13% for p24 and QRTPCR method, respectively. For MA/UF the IP/VP lies in the range of 4 - 6% and 1 - 5% for p24 and QRTPCR, respectively. Statistical analysis of the QRTPCR data showed that at high volumes there is a significant difference between MA/UF and UC, but there is no significant difference between the two purification methods at low volumes (Figure 3D). In contrast, the p24 data showed no significant difference between MA/UF and UC after purifying large volumes and significant difference between the two purification methods at low volumes (Figure 3D). Thus, there is a considerable variation and even conflicting results between the different assay methods available for the quantification of the physical titer (i. e. total number of viral particles). Nevertheless, one can conclude that the IP/VP ratios for both UC and MA/UF are in the same range. Both methods exhibited a similar efficacy in purifying biologically active viruses (see also Table 1) with a tendency of higher IP/VP ratios after UC purification.

The purity of LV preparations is an important issue, especially in clinical applications. Therefore, we analyzed MA/UF preparations for their protein (Figure 3E) and DNA content (Figure 3F). Use of DMEM with FCS for culturing the packaging cells resulted in a high concentration of proteins in the SN as well as in the concentrate presumably due to presence of serum proteins especially albumin (molecular weight: 67 kDa). Next, we tested whether serum free medium can be used for purification of LV containing SN. Two different serum-free media (HyClone SFM4MegaVir and Panserin608) were tested. These media clearly resulted in a reduction of protein concentration in the SN with HyClone SFM4MegaVir leading to the highest reduction of protein carry-over in the concentrate (Figure 3E). The IPs applied to the protein gel were in the same range (1 to 3 × 10^5 ^IPs for SN and 1 to 2 × 10^7 ^IPs for the concentrate) indicating that use of serum free medium does not influence the LV infectivity. Thus, cultivation of producer cells after transfection can be performed with serum free medium if reduction of the protein content of the LV preparations is desired.

To determine DNA contaminations in the concentrate, we analyzed samples from LV purifications on an agarose gel with and without treatment with benzonase (Figure 3F) [24]. Without treatment with benzonase (-benz, Figure 3F) high concentrations of DNA were present in the LV concentrates. In contrast, benzonase pretreatment of the SN efficiently reduces the DNA content (+benz, Figure 3F). The biological titers of the concentrates obtained with the two purifications were in the same range (-benz: 1.3 × 10^9 ^IP/ml; +benz: 1.9 × 10^9 ^IP/ml), indicating that benzonase treatment does not impair the biological activity of the LVs.

### Concentration of non-VSV.G pseudotyped lentiviral vectors

Presently, most LVs used are pseudotyped with VSV.G, mainly due to its broad host range, high infectious titers and stability during UC [1,15]. Nevertheless, for some applications other envelope proteins are required [25,26]. Furthermore, toxicity of VSV.G pseudotyped vectors as well as an induction of immune response upon systemic administration has been observed [13,27].

**Figure 4:**
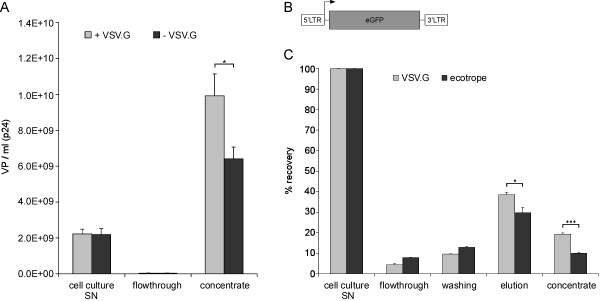
**Comparison of lenti- and retroviral vectors with different pseudotypes**. **(A) **LV production was either performed with (+) or without (-) VSV.G pseudotyping and 40 ml of the cell culture SN were purified using MA/UF and analyzed by p24-ELISA, respectively. The viral particles per ml were calculated (*n = 3, mean +SEM*). **(B) **Schematic representation of oncoretroviral vector used. eGFP, enhanced green fluorescent protein; LTR, long terminal repeat. **(C) **Oncoretroviral vectors were produced with VSV.G pseudotyping (VSV.G) or ecotropic envelope (ecotrope) and 40 ml of cell culture SN were each purified using MA/UF setup. The physical titer of the fractions was analyzed using C-type RT-activity kit and the recovery rate was calculated, respectively. n = 3, mean+SEM. * 0.05 ≥ p > 0.01; *** 0.001 ≥ p.

Therefore, we tested whether the VSV.G envelope is required for purification with the MA system. Cell culture SNs containing viral particles either with VSV.G envelope or without envelope were each purified with the small MA/UF system and samples were analyzed by p24-ELISA (Figure 4A). Similar titers in the SN (approximately 2.2 × 10^9 ^VP/ml) and nearly no viral particles were found in the flowthrough in both cases. Although significant higher titers were obtained with VSV.G pseudotyped viral particles, this experiment clearly shows that VSV.G is not required for binding to the adsorber material.

### Purification of oncoretroviral vectors

Retroviral vectors have been established from different retroviruses. They play an important role in gene therapy [1]. Among the retroviruses, oncoretroviruses represent presently the most commonly used vector type. Therefore, we tested whether the MA system can also be applied for the purification of oncoretroviral vectors. We used MoMLV-derived vectors containing an eGFP expression cassette under control of the MoMLV-LTR (Figure 4B) [28]. In initial experiments, VSV.G pseudotyped retroviral as well as VSV.G pseudotyped lentiviral particles were purified in parallel using 40 ml of cell culture SN and the small MA/UF combination, respectively. The recovery rate for the VSV.G pseudotyped retrovirus as analyzed by FACS was almost 30% (data not shown) demonstrating the utility of the MA system also for this vector system.

Although retroviruses that carry the ecotropic envelope have a narrow host range, this type of vector is also widely used [11]. Therefore, we tested the MA/UF setup for the purification of 40 ml cell culture SN containing ecotropic oncoretroviral vectors. For comparison, we additionally analyzed oncoretroviral vectors with VSV.G envelope. Samples were analyzed using a C-type RT activity kit (Figure 4C). In the elution fraction 38.5 ± 1.3% of the VSV.G pseudotyped particles and 29.8 ± 2.5% of the retroviral particles with ecotropic envelope were recovered, respectively. After the UF, the recovery rate of the ecotropic virus was 10.0 ± 0.3% whereas 19.2 ± 0.7% of the initially applied VSV.G pseudotyped particles were detected in the concentrate.

In summary, these data indicate that VSV.G is not necessary for binding to the MA and that also purification of viral particles pseudotyped with other envelope proteins is possible. Although binding to the adsorber works best with VSV.G envelope, these results demonstrate the broad application spectrum of the MA/UF system presented in this study.

## Discussion

Here, we describe a method for purification and concentration of lentiviral as well as oncoretroviral particles. Due to their capability of integrating their RNA after reverse transcription into the host genome, recombinant retroviral vectors are versatile tools in a broad spectrum of applications. LVs have become a convenient alternative to oncoretroviral vectors, because of their ability to transduce dividing as well as non-dividing cells.

Presently, the most common way of concentrating VSV.G pseudotyped viral vectors is UC. However, purification methods based on UC are difficult to scale up and time-consuming due to the limited capacity of the centrifuges. Furthermore, impurities having a similar density as the viral particles (membrane vesicles of the host cells e. g.) are coprecipitated during the centrifugation steps [29,30]. Alternatively, chromatography can be applied for vector concentration, including affinity chromatography, anion exchange and size exclusion. Chromatography is a very useful procedure to separate macromolecules like DNA, proteins and also viral vectors. But many chromatographic media are inapplicable because of the relatively labile nature of lenti- and retroviral vectors [11]. Affinity chromatography strategies are based on streptavidin-biotin interaction [31], heparin binding [32] and immobilized metal affinity [33]. In anionic exchange chromatography the negatively charged viral vectors bind to the positively charged chromatographic material. Bound virus can be then eluted by high salt buffer [11,18,34]. Elution with high salt buffers significantly reduces the infectivity of viral vectors, very likely due to an increase in osmotic pressure and a subsequent damage of the viral membrane. We found that only one hour of exposure to high salt (1 M NaCl) on ice is enough to inactivate 16.0 ± 6.4% of the LVs (Additional file 1, Figure S1). Others observed a reduction by 50% of infectious retroviral particles when exposed to high salt for one hour at room temperature [32]. Therefore, further purification steps are necessary [32]. However, anionic exchange requires no modification of the viral vectors such as biotinylation or his-tagging and is fast, versatile and easy to scale up [11,35,36]. Several anion-exchange chromatographic matrices have already been investigated with different binding capacities for retroviral particles [37]. Another chromatographic procedure is size exclusion. Although it is a very powerful separation method, it is mainly applied as a polishing step due to difficulties in up-scaling and a low throughput [11,38]. Another way to purify lenti- and retroviral vectors is UF through membranes with various pore sizes (20-500 kDa). Although UF is a fast and simple purification method, an undesirable side effect is concentration of macromolecules derived from cell debris [39]. Further alternatives for purifying lenti- and retroviral particles are precipitation with salt buffers (ammonium sulphate, calcium phosphate) and/or polymers (e.g. polyethylene glycol, poly-L-lysine). But this method has the disadvantage that the salts and polymers as well as impurities (e.g. cell debris) are co-sedimented and therefore, additional purification steps are often necessary. Furthermore, this method has a low selectivity and is difficult to scale up [11,40].

Here, we used a combination of anionic exchange and UF for the purification of VSV.G pseudotyped lenti- and retroviral particles. The virus was bound to the cationic membrane by loading the cell culture SN to the MA. Elution of the viral particles was achieved by application of high salt buffer. However, due to toxic effects of salt buffers with high molarity [32,38] subsequent rebuffering is necessary. We used UF units for desalting and concentration, leading to high titer LV preparations with a biological titer in the range of 2 to 5 × 10^9 ^IP/ml.

MAs of different capacity were applied to purify 40 ml and 500 ml of cell culture SN, respectively. The number of infectious lentiviral particles obtained in the concentrates after MA/UF was similar or even higher as compared to preparations purified with UC of the same starting volume (Additional file 4, Figure S4). Importantly, the MA/UF system allows for purification of up to 1,000 ml of starting volume containing LVs by a serial coupling of two large MAs and final UF. Furthermore, the MAs used in our study can be connected to FPLC (data not shown). This enables an automation of the purification procedure as well as monitoring of different parameters like UV extinction or conductivity.

For use of LVs in clinical trials (e. g. [41]), high titer vector preparations are needed that can be reproducibly obtained at large scale. Impurities that derive from transfection and cell debris of producer cells as well as medium components should be minimized in the LV preparations for such applications. In addition, some primary cells (e.g. primary cortical neuronal cultures) have been shown to be sensitive to toxic effects of FCS components supplemented in the medium [42]. In this context it is of interest that we observed a clear reduction of protein and DNA contaminants by use of serum free medium and after treatment of cell culture SN with benzonase, respectively (Figure 3E and 3F) [24,43].

Another important property of LV preparations is the ratio of infectious vs. total viral particles. Overall, we observed that between 1 and 20% of the purified particles were biologically active, which is similar or even better than already published IP/VP ratios (0.1 to 1%) [22,23]. These differences might be due to various protocols for LV preparation and purification procedures as well as cell lines and constructs used.

Different set ups for the cultivation of the adherent HEK 293T packaging cells have been described in the combination with MAs. For example Kutner et al published a combination of multilayered flasks with a gas permeable growth surface (called HYPERFlask vessels) and anionic exchange chromatography [35]. Cultivation of the packaging cells in the HYPERFlask vessels, instead of conventional cell culture dishes, resulted in 10 fold increased LV production [35]. Furthermore, LV generation of cells in suspension culture were recently analyzed by Lesch et al to facilitate a scalable LV preparation for gene therapy applications [44]. For this purpose, baculovirus based LV production of suspension cells was used for transduction of the packaging cells with the lentivector/packaging plasmids instead of the commonly applied transfection of adherent cells by calciumphosphate. However, in order to remove the baculoviruses a purification step had to be included [44].

It remains to be shown that these systems can also be used for particles carrying different envelopes and for vectors derived from oncoretroviruses.

An outstanding feature of the purification method described herein is that it can be applied for the purification of lenti- as well as oncoretroviral vectors. Although a direct comparison between viral vectors derived from different members of the retrovirus family is not feasible (e.g. transgene expression driven by CMV versus LTR elements in the lentiviral and retroviral vectors, respectively), the recovery rates obtained with the VSV.G pseudotyped retrovirus is in the similar range as the recovery of VSV.G pseudotyped lentiviruses.

In addition, viral particles pseudotyped with different envelopes can be purified, which broadens the spectrum of possible applications considerably. Apart from VSV.G also other glycoproteins were shown to be useful for pseudotyping lentiviral particles including envelope proteins from ross river virus, semliki forest virus, mokola virus or rabies virus [45,46]. Initially, we showed that purification of LVs is possible even without any envelope protein showing that the presence of VSV.G is not a perquisite for LV purification and opening the possibility of alternative pseudotyping. In this context is of interest that the MA/UF system can also be used to purify viral particles carrying an ecotropic envelope. However, purification of the oncoretroviral particles with VSV.G pseudotyping resulted in a significant higher recovery rate compared to ecotropic envelope protein. This difference might be due to reduced stability of ecotropic pseudotyped viral particles [11]. In general, the lenti- and retrovirus stability depends not only on the particular envelope protein but also on the producer cell line type from which the viral lipid envelope was derived [9,13] and other factors like buffers, pH, shear forces, temperature and especially presence of high salt concentrations [32,37,43].

## Conclusions

The MA/UF method described here is a versatile way to purify high titer lentiviral as well as oncoretroviral particles starting from small and large volumes. With the MAs described a broad range of starting volumes up to one liter of virus supernatant can be rapidly processed. Importantly, even at large scale/higher input volumes the method yields reproducibly high virus titers. Furthermore, the procedure is easy to handle and can even be connected to an automated purification system such as FPLC allowing for monitoring accurate salt gradient elution, which is an important factor for further use in a clinical setting. In contrast to the widely used UC method, no expensive equipment like ultracentrifuges are needed.

Importantly, this method can be used for vectors derived from different members of the large family of retroviruses independently of the envelope (pseudotyping) used. Taken together, the MA/UF combination described in this study offers a flexible and rapid way to purify lentiviral particles as well as oncoretroviral vectors with different pseudotypes from a broad range of starting volumes at reproducibly high titers.

## Methods

Unless stated otherwise, all chemicals and biochemicals used in this study are from highest purity (tested for cell culture).

### Viral vector preparation

The original virus plasmids of the 3^rd ^generation are derived from the lab of Inder Verma (The Salk Institute for Biological Studies, Laboratory of Genetics, La Jolla, CA, USA). The lentivector system consists of two major parts: the vector and the packaging constructs. The used lentivector rrl-CMV-eGFP (Figure 1A) contains a CMV-driven eGFP expression cassette. The promoter/enhancer sequences in the U3 region of the 3' LTR were deleted to generate self-inactivating (SIN) vectors [47]. The used oncoretroviral vector pCLMFG-GFP (Figure 4B) is based on MoMLV with an eGFP expression cassette driven by the MoMLV-LTR [28]. The packaging system used for LV production was described in detail previously [16]. Oncoretroviral particles were produced with the CMV-*gag*/*pol *packaging plasmid [28] and for pseudotyping either VSV.G or ecotropic envelope was used.

### Production of recombinant virus

The production of LVs was performed as described previously [21]. Briefly, HEK 293T cells were seeded on poly-L-lysine coated 150 cm^2 ^cell culture dishes (BD Falcon, Franklin Lakes, NJ, USA) or 5 chamber cell stacks (surface area in sum 3180 cm^2^; Corning Incorporated, Lowell, MA, USA) in DMEM (Invitrogen, Darmstadt, Germany), supplemented with 10% fetal calf serum (Biochrom, Berlin, Germany), 100 U/ml penicillin G/100 μg/ml streptomycin (Pen/Strep; Biochrom, Berlin, Germany) and incubated at 10% CO_2 _and 37°C. Cells were transfected at ~50% confluency by calciumphosphate transfection with 22.5 μg lentivector, 14.6 μg pMDLg/pRRE, 5.7 μg RSV-rev and 7.9 μg pMD.G (VSV.G) per 150 cm^2 ^cell culture dish. Amounts for cell factories were calculated according to the surface area (one 5 chamber cell stack is equivalent to 21.2 150 cm^2 ^cell culture dishes). For production of oncoretroviral vectors transfection was performed using 25 μg retroviral vector pCLMFG-GFP, 12.5 μg pMD.G or CMV-ecotrope and 25 μg CMV *gag*/*pol *per 150 cm^2 ^cell culture dish. For transfection, cells were cultured at 3% CO_2 _and 37°C over night. On the next morning, the medium was replaced by 20 ml fresh supplemented DMEM per 150 cm^2 ^dish and 350 ml per cell factory. For analysis of protein content medium was replaced by serum free medium (HyClone SFM4MegaVir, Thermo Fisher Scientific, Waltham, MA, USA or Panserin 608, PAN Biotech, Aidenbach, Germany), supplemented with Pen/Strep. Cells were cultured again at 10% CO_2 _and 37°C.

### Purification of recombinant virus

For purification of viral vectors, cell culture SN was collected 24 h after adding fresh medium. To remove cell debris the SN was filtered either using a bottle-top filter (SFCA, 0.45 μm, Nalgene, Thermo Fisher Scientific, Waltham, MA, USA) or the Sartopore 2 150-filter (PES, 0.45 μm-0.2 μm, Sartorius Stedim Biotech, Goettingen, Germany).

The components and buffers necessary for the purification using MA/UF are commercially available as LentiSELECT kits for different starting volumes of cell culture SN (40 ml: LentiSELECT40; 500 ml: LentiSELECT500; 1,000 ml: LentiSELECT1000). The purification procedure was performed according to the manufacturer's manual (Sartorius Stedim Biotech, Goettingen, Germany).

The UC protocol was described previously [21]. Briefly, the filtered solution was transferred to centrifugation tubes and centrifuged in an ultra-centrifuge (Optima L-100 XP, Beckman Coulter Incorporated, Brea, CA, USA) with SW32Ti rotor (Beckman Coulter Incorporated, Brea, CA, USA) for two hours at 19,400 rpm and 17°C. Subsequently, virus pellets were resuspended in HBSS (Invitrogen, Darmstadt, Germany) and combined. The pre-concentrated suspension was transferred into a centrifuge tube and layered on top of a 2 ml 20% (w/v) sucrose cushion. The tubes were centrifuged using a SW55 rotor (Beckman Coulter Incorporated, Brea, CA, USA) for 2 hours at 21,000 rpm at 17°C. The pellet was resuspended in an appropriate volume of HBSS. The tube was vortexed for 45 minutes at 1,400 rpm and 17°C, followed by a short centrifugation step (3 s, 16,000 g) to pellet debris. Finally, the opaque SN was transferred to a new tube.

For both methods, samples were taken from every purification step and used for the transduction of cells (see also analysis of viral titer) or stored at -80°C for further analysis.

### Analysis of viral titer

The biological titer of the LVs carrying the eGFP reporter was analyzed by transduction of HEK 293T cells followed by flow cytometry and IP/ml were calculated as previously described [21]. For analyzing the physical titers of the LV containing solutions either the HIV-1 p24 Antigen ELISA from Zeptometrix Corporation (Buffalo, NY, USA) was used to measure the amount of the lentiviral p24 capsid protein according to the manufacturer's instructions or QRTPCR was applied to determine the viral RNA genome [23]. For calculating the viral particles per ml (VP/ml) from p24 concentration the following term was used according to manufacturer's manual: VP/ml = pg p24/ml × 10,000. Measurements were each done from different dilutions of LV preparations. To establish a calibration curve for QRTPCR measurements the lentiviral plasmid rrl-CMV-GFP (Figure 1A) of known concentration (measured by spectrophotometry) was used and standards were generated by applying 10-fold serial dilutions. The number of DNA molecules was each determined by including molecular weight of the plasmid, Avogadro constant and dilution factor. The plasmid was amplified by QRTPCR using specific TaqMan^® ^primers for lateRT (for: 5'-TGTGTGCCCGTCTGTTGTGT-3'; rev: 5'-GAGTCCTGCGTCGAGAGAGC-3') and lateRT probe (5'-FAM-CAGTGGCGCCCGAACAGGGA-BHQ1-3'). Ct values measured were plotted against the number of plasmid DNA molecules and a standard curve was generated (Additional file 5, Figure S5). Ct values were determined at least in triplet. For measurement of samples RNA was isolated from the concentrated LV preparations by use of m*ir*Vana™ miRNA Isolation Kit according to the manufacturer's instructions (Applied Biosystems, Carlsbad, CA, USA). RNA was transcribed into cDNA using Transcriptor First Strand cDNA Synthesis Kit from Roche Diagnostics (Mannheim, Germany) according to the manual. QRTPCR was then performed with TaqMan^® ^probe and primers as described before. By using the calibration curve the viral particles per ml were calculated from the Ct values considering the diploid lentiviral RNA genome. Measurements were each done in triplet from three different dilutions of LV preparations.

In case of oncoretroviral particles the C-type RT-activity kit from Cavidi AB (Uppsala, Sweden) was used to analyze the activity of viral reverse transcriptase. Procedure was performed from different sample dilutions according to the manufacturer's manual. Reverse transcriptase activity was measured colorimetric in mu/ml.

### Protein gel electrophoresis and Western blot analysis

For analysis of protein content LV containing fractions were loaded onto SDS-protein gel and coomassie blue stained [48]. For detecting viral particles via Western blot analysis antibody against lentiviral capsid protein p24 was used. Viral particles containing fractions were applied to SDS-protein gel and immunoblotting on a PVDF-membrane was performed. Blocking of membrane was achieved with 5% (w/v) skimmed milk powder in PBS (phosphate buffered saline). Polyclonal antibody to HIV1 p24 (abcam, Cambridge, UK) was applied 1:2,000 in TBS (Tris-buffered saline) with 1% (v/v) Tween20 (TBST) for 2 h at room temperature. After washing, peroxidase-conjugated second antibody Anti-Rabbit IgG (0.8 mg/ml; dianova, Hamburg, Germany) was used 1:10,000 in TBST (1 h at room temperature). For imaging, chemiluminescent substrate for horseradish peroxidase (Thermo Fisher Scientific, Waltham, MA, USA) was applied.

### Agarose gel electrophoresis

For detection of coeluted DNA LV containing fractions were loaded onto 0.7% (w/v) agarose gel and electrophoresis was performed in 1x TBE (Tris/borate/EDTA) buffer. The gel was stained with ethidium bromide and analyzed using a gel documentation system [48].

### Electron microscopy

Images of purified viral particles were made using electron microscopy with negative staining procedure. The viral particles were mixed with 1% (v/v) glutaraldehyde solution and a droplet was placed on parafilm for 10 min. A formvar covered 200 mesh grid coated with carbon (Plano GmbH, Wetzlar, Germany) was placed on the droplet for 5 min. Remaining solution was soaked with a filter paper. The grid was then placed on a droplet of phosphotungstic acid for 30 min and excess liquid was again soaked with a filter paper. After drying, the grid was put into the transmission electron microscope Philips CM 10 with digital imaging analySiS from SIS (Olympus Europa GmbH, Hamburg, Germany) and images were taken.

## Competing interests

The study was supported by a grant from Sartorius Stedim Biotech GmbH, Goettingen, Germany, including the salary of a post-doctoral fellow during the initial phase of the study as well as lab equipment and consumables. The experiments were performed at the University of Bonn.

## Authors' contributions

KZ and JM carried out the experiments presented in this study. KZ wrote the manuscript together with AP. OS assisted in the use of the MAs and provided preliminary data concerning the MAs and the different buffers used for anionic exchange. All authors participated in the design and coordination of the study. AP, EJ and KZ conceived the study and AK contributed intellectual input. All authors read and approved the manuscript.

## Supplementary Material

Additional file 1 **Figure S1. Effect of high salt elution buffer**. High salt elution fractions were used for transduction of HEK 293T cells and thereafter incubated on ice for 0.5 h, 1 h, 1.5 h and 2 h and utilized again for infection. The biological titer was determined and the recovery was calculated in relation to IPs at the beginning, respectively. n = 3, mean +SEM. * 0.05 ≥ p > 0.01; ** 0.01 ≥ p > 0.001. Click here for file

Additional file 2 **Figure S2. Westernblot-analysis of LV purification using MA/UF combination**. 40 ml of LV containing cell culture SN was purified using MA/UF setup and 15 μl of cell culture SN (SN), elution fraction (elution) as well as concentrate (conc) were loaded on SDS-protein gel and Westernblot was performed with p24-antibody. One representative blot (*of n = 4*) is shown. Click here for file

Additional file 3 **Figure S3. Capacity of large MAs**. 500 ml **(A) **or 1,000 ml **(B) **of the same cell culture SN were purified using the large MA **(A) **or two serial connected large MA-units **(B)**. After applying the starting solution the flow through was collected in 50 ml-steps and the biological titer was determined, respectively. Click here for file

Additional file 4 **Figure S4. Comparison of MA/UF combination and UC**. Cell culture SN (40 ml or 500 ml) containing VSV.G pseudotyped LVs were purified either by using MA/UF setup (40 ml SN: *n = 7*; 500 ml SN: *n = 6*) or UC (40 ml SN: *n = 3*; 500 ml SN: *n = 3*). The biological titer of the concentrates was each determined using FACS-analysis and IPs were calculated. *** 0.001 ≥ p. Click here for file

Additional file 5 **Figure S5. Calibration curve for quantification of LVs using qRealtime PCR**. The lentiviral plasmid rrl-CMV-GFP (Figure 1A) was amplified by QRTPCR using specific TaqMan^® ^primers and probe. Plasmid concentration was determined by spectrophotometry and standards were generated by using 10-fold serial dilutions. Ct values measured were plotted against the number of plasmid DNA molecules and a standard curve was generated (y = -1.405 ln (x) + 39.747). Ct values were determined at least in triplet (mean ± SEM). Click here for file
