# Supplementary figures and images for: Highly efficient concentration of lenti- and retroviral vector preparations by membrane adsorbers and ultrafiltration
Source: BMC Biotechnol. 2011 May 20;11:55. doi: 10.1186/1472-6750-11-55 (PMC3118112; doi:10.1186/1472-6750-11-55)

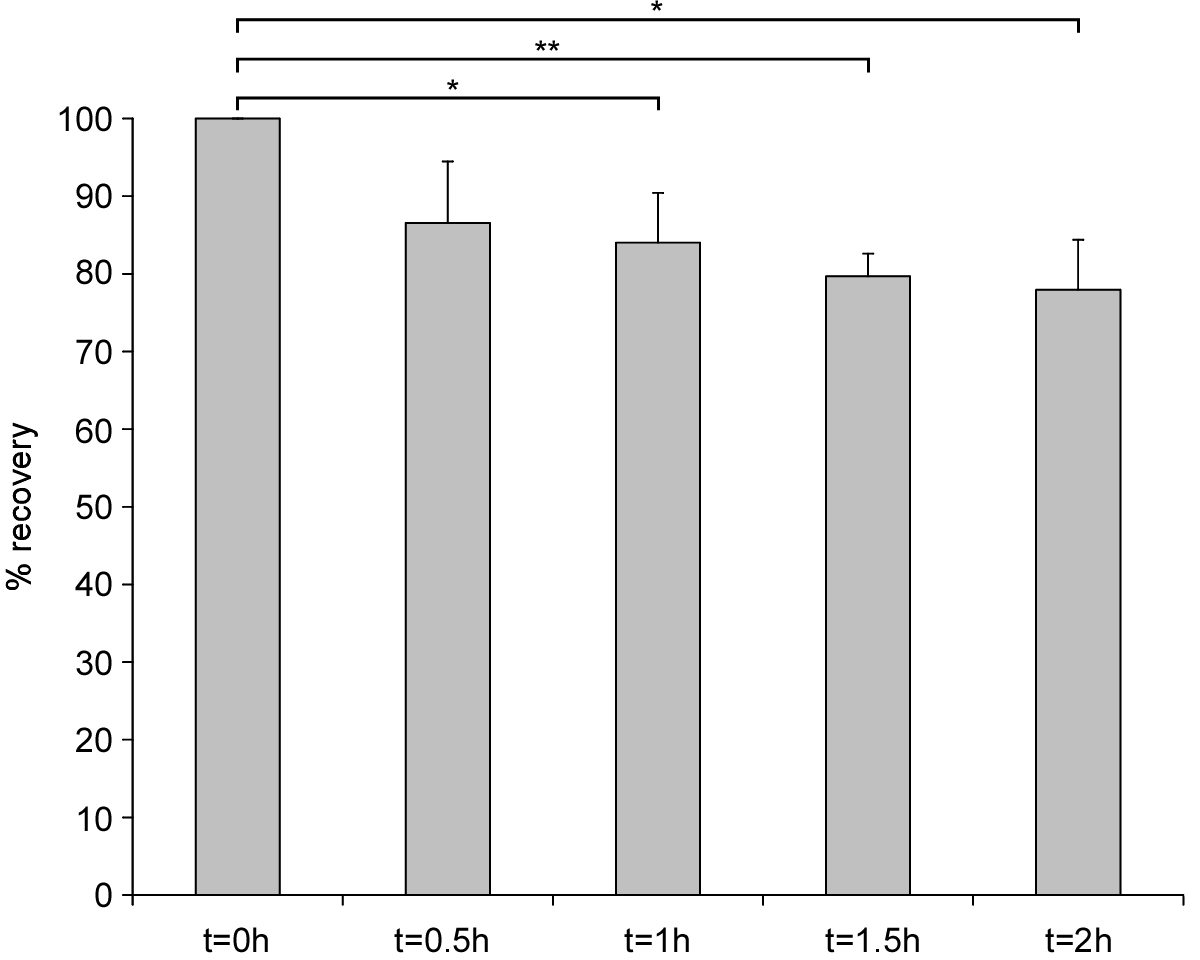

Supplement: Additional file 1 — Figure S1. Effect of high salt elution buffer. High salt elution fractions were used for transduction of HEK 293T cells and thereafter incubated on ice for 0.5 h, 1 h, 1.5 h and 2 h and utilized again for infection. The biological titer was determined and the recovery was calculated in relation to IPs at the beginning, respectively. n = 3, mean +SEM. * 0.05 ≥ p > 0.01; ** 0.01 ≥ p > 0.001. [file 1472-6750-11-55-S1.JPEG]

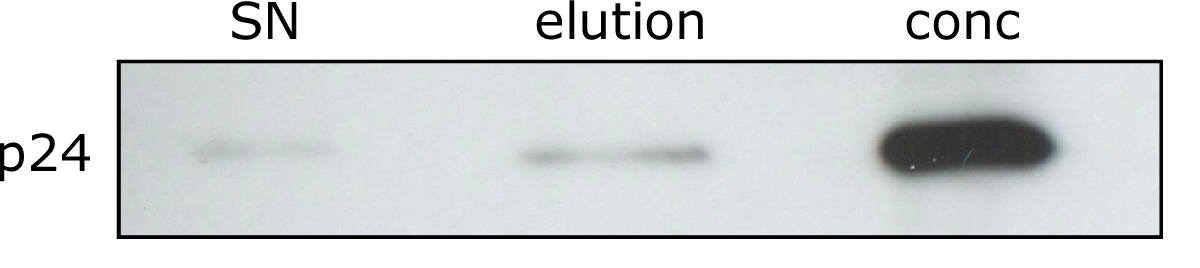

Supplement: Additional file 2 — Figure S2. Westernblot-analysis of LV purification using MA/UF combination. 40 ml of LV containing cell culture SN was purified using MA/UF setup and 15 μl of cell culture SN (SN), elution fraction (elution) as well as concentrate (conc) were loaded on SDS-protein gel and Westernblot was performed with p24-antibody. One representative blot (of n = 4) is shown. [file 1472-6750-11-55-S2.JPEG]

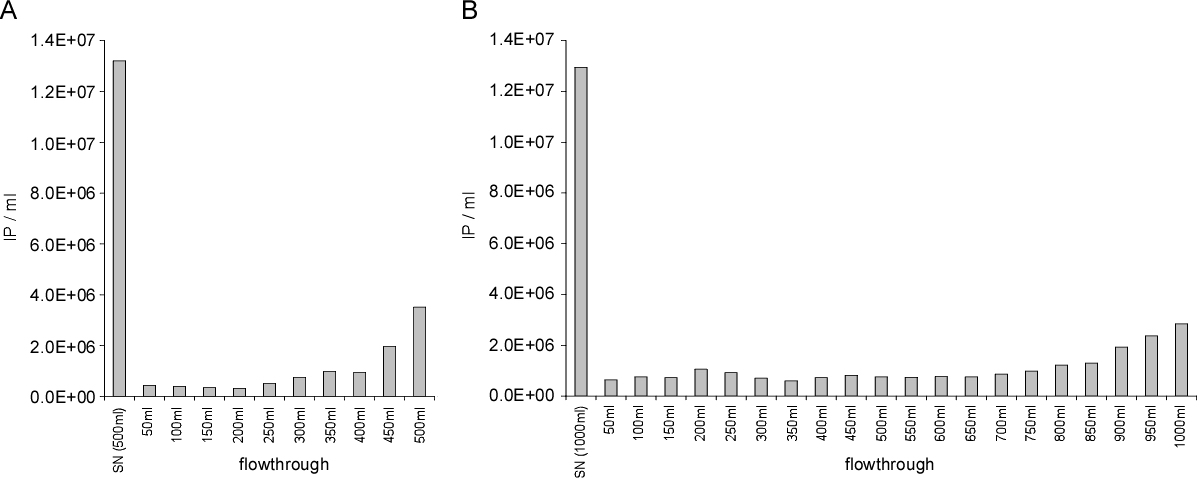

Supplement: Additional file 3 — Figure S3. Capacity of large MAs. 500 ml (A) or 1,000 ml (B) of the same cell culture SN were purified using the large MA (A) or two serial connected large MA-units (B). After applying the starting solution the flow through was collected in 50 ml-steps and the biological titer was determined, respectively. [file 1472-6750-11-55-S3.JPEG]

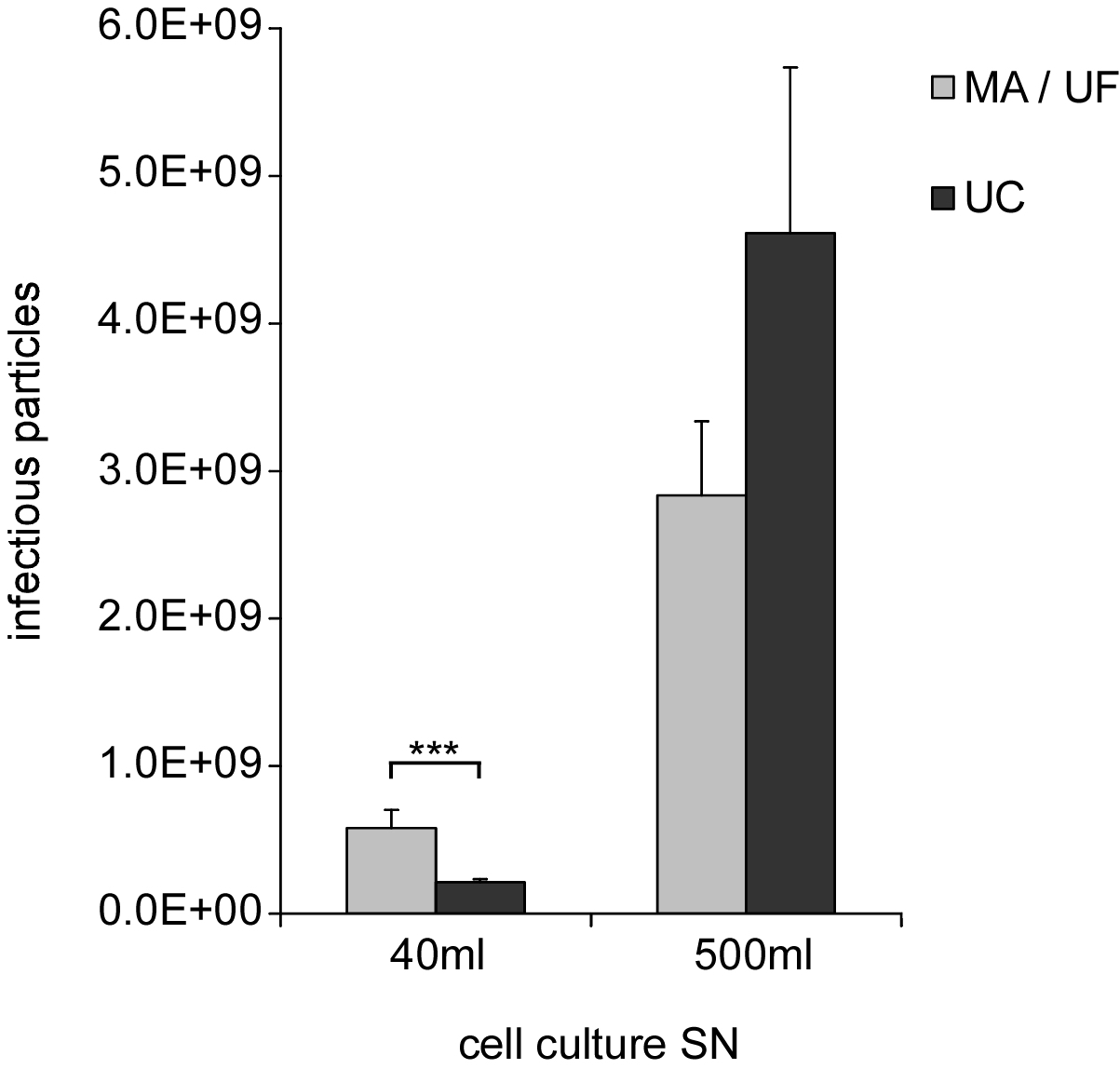

Supplement: Additional file 4 — Figure S4. Comparison of MA/UF combination and UC. Cell culture SN (40 ml or 500 ml) containing VSV.G pseudotyped LVs were purified either by using MA/UF setup (40 ml SN: n = 7; 500 ml SN: n = 6) or UC (40 ml SN: n = 3; 500 ml SN: n = 3). The biological titer of the concentrates was each determined using FACS-analysis and IPs were calculated. *** 0.001 ≥ p. [file 1472-6750-11-55-S4.JPEG]

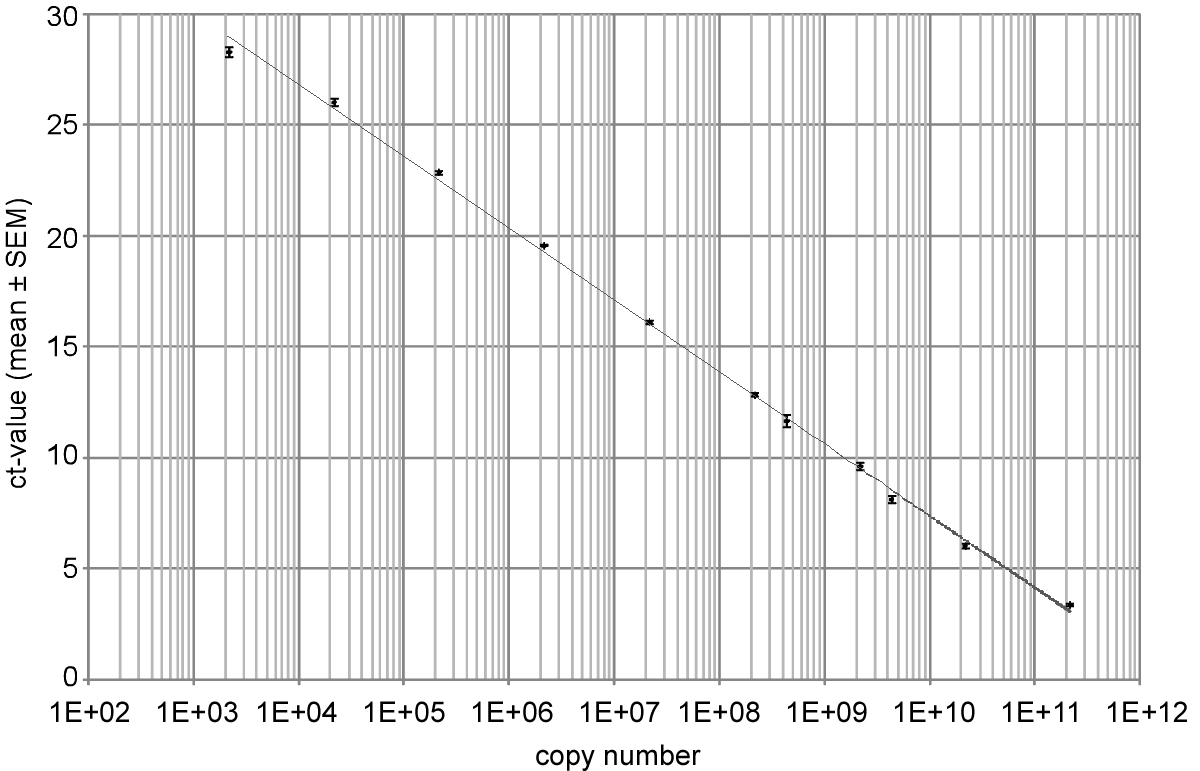

Supplement: Additional file 5 — Figure S5. Calibration curve for quantification of LVs using qRealtime PCR. The lentiviral plasmid rrl-CMV-GFP (Figure 1A) was amplified by QRTPCR using specific TaqMan® primers and probe. Plasmid concentration was determined by spectrophotometry and standards were generated by using 10-fold serial dilutions. Ct values measured were plotted against the number of plasmid DNA molecules and a standard curve was generated (y = -1.405 ln (x) + 39.747). Ct values were determined at least in triplet (mean ± SEM). [file 1472-6750-11-55-S5.JPEG]
